# Decomposing Medicare total, Part D, and Part B drug payments among people with Alzheimer's disease and related diseases

**DOI:** 10.1093/haschl/qxaf179

**Published:** 2025-09-05

**Authors:** Jie Chen, Seyeon Jang

**Affiliations:** Department of Health Policy and Management, School of Public Health, University of Maryland, College Park, MD 20742, United States; Department of Health Policy and Management, School of Public Health, University of Maryland, College Park, MD 20742, United States

**Keywords:** ADRD, Medicare spending, fee for service, CAHPS, decomposition, Part D, Part B, dual eligibility, depression, heart disease

## Abstract

**Introduction:**

This study aims to examine the extent to which health status, socioeconomic characteristics, and access to needed medications contribute to differences in total Medicare costs and drug spending among beneficiaries with and without Alzheimer's disease and related dementias (ADRD).

**Methods:**

We used Medicare fee-for-service (FFS) claims data from 2018, 2019, 2021, and 2022, linked with the Consumer Assessment of Healthcare Providers and Systems (CAHPS) survey, to examine factors associated with total Medicare spending, Part D drug spending, and Part B drug costs. Decomposition analysis was conducted to quantify the contribution of individual characteristics to observed cost differences by ADRD status.

**Results:**

Our model explained 48% of the total Medicare spending difference and 80% of the Part D drug cost gap between beneficiaries with and without ADRD. Depression, heart disease, self-reported poor health, and functional limitations were major contributors to total spending differences. Dual eligibility was a primary driver of higher Part D costs. However, the model did not adequately explain differences in Part B drug costs.

**Conclusion:**

These findings underscore the need for targeted interventions in mental health, cardiovascular care, and pharmaceutical policy. Further research is needed to better understand unmeasured drivers of Medicare spending, especially physician-administered drug costs under Part B, among beneficiaries with ADRD.

Key points

- Socioeconomic and health-related factors explained almost half of the total Medicare spending differences between beneficiaries with and without ADRD.
- Depression, heart disease, functional limitations, and dual eligibility were among the strongest factors explaining the differences in total Medicare and Part D spending between beneficiaries with and without ADRD.
- Findings support the need for targeted policy interventions in Part D coverage and chronic disease management, as well as further research to identify unmeasured drivers of physician-administered drug costs under Medicare Part B.

## Introduction

The prevalence of Alzheimer's disease and related dementias (ADRD) is projected to increase substantially in the coming decades, with the number of individuals affected expected to double and reach 13.8 million by 2060.^1^ Alongside this demographic shift, the economic burden of ADRD is also expected to rise sharply, with total annual care costs projected to grow from $360 billion in 2024 to nearly $1 trillion by 2050.^1^ At the individual level, the lifetime cost of care for a person living with ADRD is estimated at approximately $400,000, approximately 1.8 times higher than the average total lifetime Medicare spending for a typical beneficiary at age 65.^2,3^

Research has also shown that prescription medication expenses among ADRD patients are substantially elevated.^1,4^ While Part D spending has received considerable attention, Part B drug costs, such as infused or injectable medications administered in clinical settings, have been less studied, despite their growing contribution to Medicare expenditures.^5-7^ Recent approvals of dementia treatment drugs, such as lecanemab and donanemab, expected to be covered under Part B,^8,9^ have significant implications for Part B spending.^10^ Given the clinical complexity of ADRD patients and their frequent use of physician-administered therapies, it is critical to examine Part B costs in greater detail to understand their role in the overall cost burden and to ensure access to emerging therapies such as lecanemab for those who need them.

To comprehensively understand the factors driving healthcare costs and quality in this population, it is essential to integrate objective (eg, administrative claims), self-perceived health measures (eg, self-reported health), and socioeconomic status (SES) within a well-established conceptual framework. Specifically, this study aims to identify key drivers of variation in Medicare costs by comparing fee-for-service (FFS) beneficiaries with and without ADRD. Our analysis includes total Medicare payments, Part D, and Part B drug costs.

We hypothesize that the cost differences are primarily driven by coexisting chronic conditions, with mental health, particularly depression, playing a significant role. Depression has been shown to negatively affect treatment adherence, medication compliance, and overall well-being, making it a critical contributor to both increased costs and adverse health outcomes.^11-13^ In addition, we hypothesize that self-perceived health status and SES factors, such as education and health literacy, influence individuals’ ability to navigate the healthcare system, thereby affecting efficiency and costs.^(1,11,14)^

Less is known about the specific drivers of differences in drug costs between individuals with and without ADRD. People with ADRD are more likely to experience polypharmacy due to the complexity of their health needs.^15,16^ As a result, access to medications and the scope of insurance coverage play an essential role in their care management. In the health economics literature, appropriate drug treatment and medication adherence are often considered cost-effective strategies.^(17,18)^ In this study, we aim to explore the extent to which health status, socioeconomic characteristics, and access to needed medications contribute to drug cost differences, particularly among those with ADRD. Although our study does not include data on specific high-cost Part B drugs, it offers valuable insights into current patterns of Part B drug utilization among Medicare FFS beneficiaries, including those with ADRD.

## Methods

### Data

The primary dataset for this study is the Centers for Medicare & Medicaid Services (CMS) Medicare Beneficiary Summary File (MBSF). Using the beneficiary ID, we linked the MBSF with the 2018, 2019, and 2021 Medicare FFS Consumer Assessment of Healthcare Providers and Systems (CAHPS) survey.^19,20^ Data collection for 2020 was suspended due to the COVID-19 pandemic, making data from that year unavailable. The CAHPS survey is an annual survey conducted with a subset of Medicare beneficiaries enrolled in FFS. This survey collects information on beneficiaries’ experiences with Medicare and their providers, including SES and self-reported health status.^19^ Previous studies have used this dataset to test the association between patient self-perceptions with various outcomes.^(21,22)^ Using the beneficiary location ID, we linked the Social Vulnerability Index (SVI) as a proxy for the availability of local resources and the social determinants of health affecting each beneficiary.^23^

The MBSF contains detailed enrollment information for beneficiaries across Medicare Parts A, B, C, and D. It is linked with the Chronic Conditions and Cost & Use file segments, which offer data on diagnosed chronic conditions as well as summaries of healthcare utilization and total annual Medicare payments. Individuals with ADRD were identified using algorithms developed by the CMS Chronic Conditions Data Warehouse.^24^ A beneficiary was classified as having ADRD if they had at least 1 claim, across inpatient, skilled nursing facility (SNF), home health, Part B institutional, or Part B noninstitutional services, with any of the ADRD-related ICD-10 codes.^24,25^ Our study focused on Medicare FFS beneficiaries aged 65 and above.

### Outcome measures

The first primary outcome measure is the annual total Medicare payment per individual, consistent with the Medicare payment metric applied in the 2021 Alzheimer's Disease Facts and Figures Special Report.^26^ We calculated total Medicare payments per person per year by summing Medicare expenditures across key healthcare services.^26^ We also examined Part D and Part B drug Medicare payments separately. To account for inflation, all payments were adjusted to 2023 US dollars using the Consumer Price Index.^27^

### Independent variables

The covariates included in this study were derived from the Andersen behavioral model and existing literature on health services research on aging and ADRD.^4,28,29^ These independent variables capture demographic, socioeconomic, and health-related factors that influence Medicare expenditures and healthcare quality.

Key demographic variables included age categories (65-74, 75-84, and 85 and older), sex (male or female), and race/ethnicity (White, Black, Hispanic, Native American, Asian, unknown, or other) developed using the Research Triangle Institute algorithm.^30^ Enabling variables included full 12-month dual eligibility for Medicare and Medicaid (yes or no), education level (less than high school vs high school or more),^31^ ease of obtaining needed medications,^28^ and social vulnerability (above the median which represents disadvantaged areas vs below the median).^25,32-34^

Health-need covariates captured the presence of major chronic conditions commonly comorbid with ADRD, including heart disease, asthma, depression, diabetes, hyperlipidemia, and hypertension.^1,35^ We also controlled for self-reported general health and limitations in activities of daily living (ADLs) as indicators of functional status.^36^ Finally, year-fixed effects were controlled for to adjust for time-based variations in healthcare policies and macro market trends.

### Analysis

We first presented the summary statistics for individuals with and without ADRD. Next, we conducted generalized linear model (GLM) regression analyses to examine total Medicare costs, Part D, and Part B drug costs. The GLM was selected to account for the skewed distribution of cost data.^37^ Specifically, we used a log link and gamma distribution, which have been widely applied in healthcare cost analyses to address data skewness.^37^

Following the GLM regressions, we applied the Oaxaca decomposition method to compare cost differences based on ADRD status, decomposing the differences into contributions from health needs and other explanatory measures. The Oaxaca decomposition approach has been extensively used to explain group differences in various fields,^38,39^ including health services research.^11,40,41^ For example, it has been applied to assess how differences in the availability of healthcare resources, technology infrastructure, and service accessibility contribute to urban–rural disparities in healthcare quality.^40^ Additionally, the method has been used to examine how social determinants of health contribute to racial and ethnic disparities in healthcare outcomes and access.^11^

In our study, we compared outcomes of interest based on the presence of ADRD. Using cost as an example, we first estimated separate cost regressions for individuals with and without ADRD. We then decomposed the 2 regression models and rearranged the terms to distinguish between differences attributable to observed and unobserved factors. The portion of the cost difference explained by average differences in characteristics between the 2 groups reflects the contribution of observed factors. The remaining unexplained difference is attributed to unobserved factors. Additionally, we calculated the individual contributions of each controlled variable among the observed factors.

Extensive sensitivity analyses were conducted to evaluate the robustness of our findings. These included alternative model specifications, stepwise inclusion of covariates, and different estimation strategies. Results from these analyses were consistent with the main findings and are available upon request. Survey weights were applied throughout to ensure national representativeness. This study was approved by the University of Maryland’s Institutional Review Board. All analyses were performed using Stata 18 MP4.

## Results

We identified 182 003 Medicare FFS beneficiaries without ADRD and 13 435 beneficiaries with a diagnosis of ADRD. All beneficiaries were aged 65 and older. Only 4% of the sample had any SNF stays, and among those, most lasted 5 days or fewer, with only 1 case extending to 10 days. Therefore, our sample primarily represents community-dwelling older adults.

Individuals with ADRD had significantly higher costs across all spending categories (*P* < 0.001). Specifically, the average total Medicare cost for ADRD beneficiaries was $31 785, compared to $11 230 for those without ADRD. Part D spending averaged $2696 per person among individuals with ADRD vs $1687 among those without (*P* < 0.001). Similarly, Part B drug costs averaged $955 per person for ADRD beneficiaries, compared to $785 for those without ADRD (*P* < 0.001).

Summary statistics also showed that 81% of individuals with ADRD were aged 75 and older, compared to 46% among those without ADRD (*P* < 0.001). Alzheimer’s disease and related dementia patients also had higher rates of chronic conditions, including heart disease, diabetes, hypertension, and hyperlipidemia. The prevalence of depression was substantially higher among those with ADRD, 35% compared to 12% among non-ADRD beneficiaries (*P* < 0.001). Additionally, 45% of ADRD patients reported fair or poor health, more than double the rate among those without ADRD (20%, *P* < 0.001). Functional limitations were also more common in the ADRD group, with 63% reporting at least 1 ADL limitation, compared to 27% in the non-ADRD group (*P* < 0.001).

The GLM regression results for cost outcomes are presented in Table 2, adjusting for patient health needs, enabling factors, and both subjective and objective measures. Individuals with ADRD had significantly higher total Medicare costs compared to those without ADRD, with a coefficient of 0.44. This means that holding all other variables constant, having ADRD is associated with a 0.44 unit increase in the log of expected Medicare costs. Further calculation of marginal effects indicates that this increase corresponds to an average cost difference of approximately $6108.73 (*P* < 0.001).

Older adults were generally associated with higher costs, while Hispanic (coef = −0.24, *P* < 0.001), Black (coef = −0.15, *P* < 0.001), and Asian (coef = −0.43, *P* < 0.001) beneficiaries had lower overall costs. Racial and ethnic differences in Part D drug costs were generally not significant, except for Asian beneficiaries, who had significantly lower Part D drug costs (coef = −0.31, *P* < 0.001). The presence of major chronic conditions, including heart disease, depression, hypertension, hyperlipidemia, and asthma, was significantly associated with higher total costs and Part D and Part B drug costs. Self-reported fair or poor health and the presence of any ADL limitation were also strongly associated with higher healthcare costs. In contrast, individuals who reported ease of accessing prescription medications had significantly lower total and Part D drug costs (coef = −0.06, *P* < 0.001; coef = −0.23, *P* < 0.001, respectively). Costs in 2021 were higher for total, Part D, and Part B drugs Medicare spending compared to 2018 (coef = 0.06, *P* < 0.001; coef = 0.19, *P* < 0.001; coef = 0.19, *P* < 0.001, respectively).

Decomposition results of total Medicare costs are presented in Table 3. The predicted average cost for individuals with ADRD was $39 845, compared to $11 918 for those without ADRD (*P* < 0.001). Our empirical model, which included all control variables, explained $13 407 of the total cost difference of $27 927, accounting for approximately 48% of the gap (*P* < 0.001).

We further calculated the percentage contribution of each controlled variable to the total explained difference in Medicare costs and reported those contributing more than 5%. Among the covariates, heart disease, depression, self-reported fair or poor health, ADL limitations, and dual eligibility each explained a significant portion of the cost gap between individuals with and without ADRD. Specifically, heart disease accounted for 37.6% of the explained difference (*P* < 0.001), and depression contributed 18.4% (*P* < 0.001). As shown in Table 1, individuals with ADRD had significantly higher rates of heart disease and depression than those without, helping to explain the higher overall Medicare costs in this population.

**Table 1.** Descriptive characteristics of Medicare FFS beneficiaries by ADRD status.

	NO ADRD	ADRD	
	*N* = 182 003	*N* = 13 435	
	Mean (SD)	Mean (SD)	*P*
Total Medicare payment ($)	11 230.29 (25,168.76)	31 784.61 (45 185.61)	<0.001
Part D Medicare payment ($)	1686.78 (11 148.85)	2696.33 (11 445.36)	<0.001
Part B Drug Medicare payment ($)	785.49 (6218.34)	954.98 (6833.07)	<0.001
*Independent variables (0/1)*			
Age 65-74	0.54 (0.50)	0.19 (0.39)	<0.001
Age 75-84	0.35 (0.48)	0.40 (0.49)	<0.001
Age 85 or older	0.11 (0.31)	0.41 (0.49)	<0.001
Female	0.52 (0.50)	0.56 (0.50)	<0.001
White	0.85 (0.36)	0.85 (0.36)	0.230
Black	0.05 (0.22)	0.06 (0.24)	<0.001
Hispanic	0.04 (0.19)	0.05 (0.21)	<0.001
Native	0.01 (0.08)	0.01 (0.08)	0.950
Asian	0.02 (0.15)	0.02 (0.16)	0.810
Unknown race	0.02 (0.15)	0.01 (0.09)	<0.001
Other race	0.01 (0.09)	0.01 (0.09)	0.080
Heart disease	0.13 (0.34)	0.40 (0.49)	<0.001
Diabetes	0.25 (0.43)	0.36 (0.48)	<0.001
Depression	0.12 (0.33)	0.35 (0.48)	<0.001
Hypertension	0.59 (0.49)	0.79 (0.41)	<0.001
Hyperlipidemia	0.53 (0.50)	0.63 (0.48)	<0.001
Asthma	0.05 (0.21)	0.06 (0.24)	<0.001
Self-reported health: poor or fair	0.20 (0.40)	0.45 (0.50)	<0.001
Had any ADL limit	0.27 (0.44)	0.63 (0.48)	<0.001
Education level: no high school degree	0.32 (0.47)	0.46 (0.50)	<0.001
Dually eligible	0.05 (0.22)	0.12 (0.33)	<0.001
Always easy to access medicine needed	0.80 (0.40)	0.77 (0.42)	<0.001
Social Vulnerability Index: above median	0.49 (0.50)	0.52 (0.50)	<0.001
Year 2018	0.37 (0.48)	0.37 (0.48)	0.080
Year 2019	0.33 (0.47)	0.32 (0.47)	0.390
Year 2021	0.31 (0.46)	0.30 (0.46)	0.330

Source: Authors' analysis of data from the CMS Medicare Beneficiary Summary File (MBSF) linked with the 2018, 2019, and 2021 Medicare FFS Consumer Assessment of Healthcare Providers and Systems (CAHPS) Survey.

Notes: A beneficiary was classified as having ADRD if they had at least 1 claim, across inpatient, skilled nursing facility (SNF), home health, Part B institutional, or Part B noninstitutional services, with any of the following ICD-10 codes: F01.50, F01.51, F02.80, F02.81, F03.90, F03.91, F04, F05, F06.1, F06.8, G13.8, G30.0, G30.1, G30.8, G30.9, G31.01, G31.09, G31.1, G31.2, G94, R41.81, or R54. Total Medicare payments per person per year were calculated by summing Medicare expenditures across key healthcare services, including acute inpatient care, other inpatient hospital services, skilled nursing facilities, hospice care, home health services, hospital outpatient care, ambulatory surgery centers, anesthesia, Part B drugs, evaluation and management, Part B physician services, other procedures, imaging, tests, other Part B carrier services, and Part D Medicare payments. To account for inflation, all payments were adjusted to 2023 US dollars using the Consumer Price Index.

Abbreviations: ADL, activities of daily living; ADRD, Alzheimer’s disease and related dementias; FFS, fee-for-service.

Our model explained 80% of the Part D drug cost difference between individuals with and without ADRD (explained difference: $2031 vs total difference: $2543.20, *P* < 0.001). Among the covariates, self-reported health status and ADL limitations contributed 37.5% to the observed differences (*P* < 0.001), followed by heart disease at 18.4% (*P* < 0.001). Most notably, dual eligibility accounted for the largest share, explaining 42.7% of the Part D cost gap (*P* < 0.001). As shown in Table 1, dual enrollment was more common among individuals with ADRD (12%) than those without ADRD (5%). Additionally, as indicated in Table 2, dual eligibility was significantly associated with higher Part D drug costs among individuals with ADRD (coef = 1.53, *P* < 0.001).

**Table 2.** Generalized linear model results for annual per-person Medicare payments.

	Total Medicare payment		Part D Medicare payment		Part B drug Medicare payment	
	coef.	*P*	coef.	*P*	coef.	*P*
ADRD (ref: no ADRD)	0.44	<0.001	0.11	0.120	−0.10	0.170
Age (ref: Age 65-74)						
Age 75-84	0.12	<0.001	−0.05	0.250	0.33	<0.001
Age 85 or older	0.05	<0.001	−0.37	<0.001	0.30	<0.001
Female	0.02	0.210	−0.03	0.480	0.12	<0.001
Race/ethnicity (ref: White)						
Black	−0.15	<0.001	−0.06	0.560	−0.34	<0.001
Hispanic	−0.24	<0.001	−0.18	0.080	−0.29	<0.001
Native	0.04	0.540	−0.20	0.360	−0.74	0.010
Asian	−0.43	<0.001	−0.31	<0.001	−0.37	<0.001
Unknown race	−0.10	0.020	−0.28	0.010	−0.07	0.680
Other race	−0.16	0.010	−0.22	0.200	−0.41	0.010
Heart disease	0.74	<0.001	0.34	<0.001	0.15	<0.001
Diabetes	0.13	<0.001	0.42	<0.001	0.08	0.090
Depression	0.47	<0.001	0.22	<0.001	0.18	<0.001
Hypertension	0.58	<0.001	0.34	<0.001	0.45	<0.001
Hyperlipidemia	0.33	<0.001	0.09	0.050	0.24	<0.001
Asthma	0.50	<0.001	0.55	<0.001	0.57	<0.001
Self-reported health: poor or fair	0.50	<0.001	0.79	<0.001	0.81	<0.001
Had any ADL limit	0.27	<0.001	0.20	<0.001	0.08	0.070
Education level: no high school degree	−0.14	<0.001	−0.18	<0.001	−0.16	<0.001
Dually eligible	0.43	<0.001	1.53	<0.001	−0.61	<0.001
Always easy to access medicine needed	−0.06	<0.001	−0.23	<0.001	0.00	0.920
Social Vulnerability Index: above median	−0.03	0.010	0.01	0.790	0.00	0.980
Survey year (ref: year 2018)						
Year 2019	0.03	0.050	0.12	0.010	0.05	0.280
Year 2021	0.06	<0.001	0.19	<0.001	0.19	<0.001
Constant	8.17	<0.001	6.62	<0.001	5.72	<0.001

Source: Authors' analysis of data from the CMS Medicare Beneficiary Summary File (MBSF) linked with the 2018, 2019, and 2021 Medicare FFS Consumer Assessment of Healthcare Providers and Systems (CAHPS) Survey.

Notes: Coefficients are from the generalized linear model with log-link function and gamma distribution and represent the changes in the log of expected total costs. Using the coefficient of ADRD as an example, individuals with ADRD had significantly higher total Medicare costs compared to those without ADRD, with a coefficient of 0.44. This means that holding all other variables constant, having ADRD is associated with a 0.44 unit increase in the log of expected Medicare costs. Further calculation of marginal effects indicates that this increase corresponds to an average cost difference of approximately $6108.73 (*P* < 0.001).

Abbreviations: ADL, activities of daily living; ADRD, Alzheimer’s disease and related dementias; ref, reference group.

**Table 3.** Decomposition of Medicare payment differences by ADRD status.

	Total Medicare payment		Part D Medicare payment		Part B drug Medicare payment	
	Coef	*P*	Coef	*P*	Coef	*P*
ADRD $	39845.19	<0.001	4588.91	<0.001	1188.98	<0.001
No ADRD $	11918.32	<0.001	2045.71	<0.001	821.21	<0.001
Difference $	27926.88	<0.001	2543.20	<0.001	367.77	<0.001
Total explained $	13406.77	<0.001	2030.97	<0.001	462.68	<0.001
*Individual factor*	%	*P*	%	*P*	%	*P*
Heart disease	37.60	<0.001	18.40	<0.001		
Diabetes			7.06	<0.001		
Depression	18.44	<0.001	9.44	<0.001		
Hyperlipidemia	5.88	<0.001				
Self-reported health: poor or fair	17.80	<0.001	29.80	<0.001	11.03	<0.001
Had any ADL limit	10.76	<0.001	7.68	<0.001		
Dual eligible	7.68	<0.001	42.67	<0.001		

Source: Authors' analysis of data from the CMS Medicare Beneficiary Summary File (MBSF) linked with the 2018, 2019, and 2021 Medicare FFS Consumer Assessment of Healthcare Providers and Systems (CAHPS) Survey. We further calculated the percentage contribution of each controlled variable to the total explained difference in Medicare costs and reported those contributing more than 5%.

Abbreviations: ADL, activities of daily living; ADRD, Alzheimer’s disease and related dementias.

However, our empirical model did not explain the Part B drug Medicare payment difference as expected. The observed cost difference by ADRD status was $367.80, but the model overexplained this difference. In other words, if all factors were held constant, individuals with ADRD were expected to incur approximately $94.91 more in Part B drug payments than observed. Among all factors, self-reported poor health contributed to 11% of the observed difference (*P* < 0.001).

## Discussion

High healthcare costs are a major concern for individuals with ADRD, who often require extensive medical care.^1,22^ These patients typically face complex health needs and carry a disproportionate burden of other high-cost chronic conditions, such as diabetes and cardiovascular disease.^(1,4,35)^ Our findings reinforce this pattern, demonstrating that both health needs and SES significantly contribute to elevated Medicare costs among older adults, with health-related factors accounting for most of the cost burden. Among chronic conditions, heart disease and depression emerged as the most substantial contributors to the overall difference in Medicare total spending between individuals with and without ADRD.

The results of our study align with existing evidence showing that depression is highly prevalent among older adults and frequently underdiagnosed or inadequately treated in individuals with cognitive impairment.^42,43^ Depression can impair an individual's ability to engage in care management, adhere to medications, and maintain physical activity, leading to increased hospitalizations.^12,13,44^ Compromised cognitive abilities, as well as, stigma, shame, and fear about the disease, lead to delaying or forgoing needed medical care among older adults with ADRD.^45^ Depression can further deepen their disengagement from healthcare, thereby increasing the risk of unmet medical needs and worsened health outcomes. Importantly, our study found that the presence of depression was significantly associated with increased Medicare drug costs. This aligns with existing literature indicating that depression is a major risk factor for noncompliance with medical treatment and poor self-management.^44,46^ Studies have shown that depressed patients are up to 3 times more likely to be noncompliant with prescribed treatment plans compared to their nondepressed counterparts,^44,46^ potentially requiring more medications to manage deteriorated conditions.

Similarly, heart disease is a well-established risk factor for cognitive decline and the progression of dementia.^1,47,48^ Impaired blood flow can damage brain cells, leading to cognitive problems.^1,47,48^ A history of coronary heart disease has been associated with a 27% and heart failure with a 60% increased risk of dementia.^48^ Its presence typically signals greater clinical complexity, often requiring more intensive and coordinated management across outpatient and inpatient settings. The relationship between cardiovascular health and ADRD has gained increasing attention, as emerging research suggests that effective management of cardiovascular risk factors, such as hypertension, diabetes, and hyperlipidemia, early in adulthood or midlife may help reduce the risk of later-life cognitive decline.^1,48,49^

These chronic conditions not only complicate medical management but may also accelerate cognitive and functional decline in individuals with dementia,^1,35,50^ further increasing the intensity and duration of care required. Our findings underscore the critical importance of early identification and intervention targeting both mental health and cardiovascular risk factors. Investing in prevention and integrated care approaches may help mitigate future healthcare utilization and costs among older adults with or at risk for ADRD.

The results also showed that self-reported measures of health were also important predictors of healthcare spending.^51,52^ Poor or fair self-rated health was consistently associated with higher total costs.^51,52^ Those who rate their health as fair or poor are more likely to have a higher comorbidity burden and lower SES, all of which could have contributed to increased total and drug spending.^41,53^ These subjective measures, while often underutilized in claims-based models, offer critical insight into patient well-being and perceived care quality, particularly for older adults managing multiple chronic conditions and functional limitations.

We also observed that individuals with lower educational attainment had lower overall Medicare spending, which may reflect underutilization or limited access to necessary outpatient or preventive care.^54-56^ It also highlights the need for resource redistribution to provide cost-effective care delivery across socioeconomic groups.

Limitations in ADLs were another key driver of increased healthcare costs.^57,58^ Functional impairment among older adults often leads to greater reliance on acute care services, prolonged hospital stays, and increased demand for long-term care.^58^ These findings point to the broader implications of ADL limitations, not only as a marker of disease severity but also as a signal for the increased need for supportive services in the community.

While newly approved Alzheimer's treatments, such as lecanemab and donanemab, are provided through Medicare Part B,^8^ Part D covers a wide range of prescription medications that help manage ADRD-related symptoms and comorbidities, contributing to higher drug costs among older adults with ADRD.^1,4^ For prescription Part D drug spending, dual eligibility played a particularly important role. Our study found that dually eligible beneficiaries, those enrolled in both Medicare and Medicaid, were associated with substantially higher Medicare drug expenditures. As shown in Table 1, dual enrollment was more common among those with ADRD (12%) compared to those without ADRD (5%). The difference in dual enrollment rates helps explain the higher Medicare payments for Part D drugs among ADRD patients, reflecting the complex health needs and intensive medication use among this population. In addition, dual enrollment provides significant assistance with Part D out-of-pocket costs,^59^ which may further contribute to the observed spending difference.

In addition to total and Part D drug spending, we also examined Part B drug costs, which include physician-administered medications such as injectable therapies, infused drugs, and biologics often used in clinical settings such as outpatient hospitals or physician offices. Part B medications are increasingly used in the management of chronic and complex conditions, including cancer, autoimmune disorders, and advanced cardiovascular disease.^7,60^ In our study, Part B drug costs were higher among ADRD patients, likely reflecting the increased clinical complexity and comorbidity burden in this population. These drugs often have a high cost and require ongoing administration, contributing to the growing share of Medicare expenditures.^5,10^ For example, the annual price of lecanemab (Leqembi) is $26 500, and it may become one of the top-spending Medicare Part B drugs depending on the take-up.^61^ Ensuring appropriate use, improving site-of-care efficiency, and coordinating care around specialty drug management may be critical to controlling costs while maintaining access to necessary treatments.

Our decomposition model explained a substantial portion of the observed differences in Medicare costs between ADRD and non-ADRD patients. However, a significant share of the variation in spending remains unexplained. These gaps highlight the need for future research to explore unobserved factors such as disease severity, cognitive function, patient preferences, and additional patient-reported measures of quality and access. Understanding these dimensions will be essential to developing more precise models and effective interventions tailored to the needs of individuals with ADRD.

Our study has several limitations. First, the cross-sectional design restricts our ability to draw causal inferences. While we identified associations between self-reported quality measures and healthcare costs, these findings should not be interpreted as evidence of causality. Second, our analysis focused exclusively on Medicare FFS beneficiaries, who may differ from those enrolled in Medicare Advantage or stand-alone Prescription Drug Plans in terms of demographics and clinical characteristics. Additionally, our study was limited to community-dwelling beneficiaries. As such, our findings may not be generalizable to institutionalized populations or individuals with more advanced dementia. Third, we relied on CAHPS survey data linked to Medicare claims to assess healthcare patterns. However, the CAHPS survey may underrepresent individuals with more severe ADRD due to lower response rates and the exclusion of proxy respondents, introducing potential selection bias. In our linked dataset, the prevalence of ADRD was approximately 7%, slightly below the national estimate of 10% among adults aged 65 and older.^1^ Additionally, data collection was interrupted in 2020 due to the COVID-19 pandemic, which may limit comparability over time. Finally, we were unable to adjust for ADRD severity or incorporate proxy responses in the analysis due to data limitations. These constraints may affect the interpretation of patient-reported measures and overall findings.

## Policy implication and conclusion

Our findings highlight the importance of addressing both health-related and socioeconomic factors to reduce Medicare spending associated with ADRD. The substantial cost differences between individuals with and without ADRD reflect the complex and sustained care needs of this population. Among the most influential contributors to these differences were mental health conditions, especially depression, and cardiovascular diseases.

The connection between heart health and brain health is well established in the literature.^47-49^ Conditions such as hypertension, heart disease, and stroke not only contribute significantly to medical costs but are also linked to an increased risk of cognitive decline and dementia.^1,48,49^ Mental health is also a critical component of dementia care.^13,44^ Expanding access to geriatric mental health services and promoting early detection and treatment of depression may help reduce avoidable healthcare utilization and improve the quality of life for older adults with ADRD. Preventive strategies that promote healthy lifestyle behaviors,^1,62^ including regular physical activity and strong social connections, should be emphasized across the life course to help delay the onset and progression of both cardiovascular and cognitive conditions.

Further research is needed to better understand the implications of high-cost medications, particularly among dually eligible beneficiaries who face greater clinical complexity and significantly higher drug-related Medicare spending. While improving access to medications remains a critical policy goal, careful attention is warranted when interpreting self-reported measures, such as ease of accessing prescription medications. Individuals who are prescribed fewer or lower-cost medications may report greater ease of access while also incurring lower overall spending. This relationship may confound the association between perceived access and actual drug costs.

Our decomposition model explained less of the difference in Part B drug costs between ADRD and non-ADRD patients, indicating a need for further investigation into the use and drivers of physician-administered medications in this population. These findings underscore the importance of better understanding Medicare's Part B pricing structure, particularly how it affects access and spending among high-need populations, such as those with ADRD. This insight is increasingly relevant as more specialty drugs targeting age-related and neurodegenerative conditions enter the market.^7,61^ Our results may inform future evaluations of emerging therapies. It is essential to assess the broader implications of drug coverage and pricing on Medicare's long-term sustainability.

## Supplementary Material

qxaf179_Supplementary_Data
